# Contrast-enhanced MRI-based intratumoral heterogeneity assessment for predicting lymph node metastasis in resectable pancreatic ductal adenocarcinoma

**DOI:** 10.1186/s13244-025-01956-0

**Published:** 2025-03-30

**Authors:** Junjian Shen, Qing Li, Lei Li, Tianyu Lu, Jun Han, Zongyu Xie, Peng Wang, Zirui Cao, Mengsu Zeng, Jianjun Zhou, Tianzhu Yu, Yaolin Xu, Haitao Sun

**Affiliations:** 1 Department of Radiology, Zhongshan Hospital, Fudan University, Shanghai Institute of Medical Imaging, Shanghai, China; 2 Department of Radiology, Fengyang County People’s Hospital, Chuzhou, China; 3 Department of Radiology, The First Hospital of Jiaxing, Affiliated Hospital of Jiaxing University, Jiaxing, China; 4 Department of Radiology, The First Affiliated Hospital of Bengbu Medical University, Bengbu, China; 5 Department of Radiology, Affiliated Hospital of Jiangnan University, Wuxi, P.R. China; 6 Department of Research Center, Shanghai United Imaging Intelligence Co., Ltd., Shanghai, China; 7 Department of Radiology, Zhongshan Hospital (Xiamen), Fudan University, Xiamen Municipal Clinical Research Center for Medical Imaging, Fujian Province Key Clinical Specialty for Medical Imaging, Xiamen Key Laboratory of Clinical Transformation of Imaging Big Data and Artificial Intelligence, Xiamen, China; 8 Department of Interventional Radiology, Zhongshan Hospital, Shanghai, China; 9 Department of Pancreatic Surgery, Zhongshan Hospital, Fudan University, Shanghai, China

**Keywords:** Pancreatic ductal adenocarcinoma, Habitat, Magnetic resonance imaging, Lymph node metastasis

## Abstract

**Objectives:**

To develop and validate a contrast-enhanced MRI-based intratumoral heterogeneity (ITH) model for predicting lymph node (LN) metastasis in resectable pancreatic ductal adenocarcinoma (PDAC).

**Methods:**

Lesions were encoded into different habitats based on enhancement ratios at arterial, venous, and delayed phases of contrast-enhanced MRI. Habitat models on enhanced ratio mapping and single sequences, radiomic models, and clinical models were developed for evaluating LN metastasis. The performance of the models was evaluated via different metrics. Additionally, patients were stratified into high-risk and low-risk groups based on an ensembled model to assess prognosis after adjuvant therapy.

**Results:**

We developed an ensembled radiomics–habitat–clinical (RHC) model that integrates radiomics, habitat, and clinical data for precise prediction of LN metastasis in PDAC. The RHC model showed strong predictive performance, with area under the curve (AUC) values of 0.805, 0.779, and 0.615 in the derivation, internal validation, and external validation cohorts, respectively. Using an optimal threshold of 0.46, the model effectively stratified patients, revealing significant differences in recurrence-free survival and overall survival (OS) (*p* = 0.004 and *p* < 0.001). Adjuvant therapy improved OS in the high-risk group (*p* = 0.004), but no significant benefit was observed in the low-risk group (*p* = 0.069).

**Conclusion:**

We developed an MRI-based ITH model that provides reliable estimates of LN metastasis for resectable PDAC and may offer additional value in guiding clinical decision-making.

**Critical relevance statement:**

This ensemble RHC model facilitates preoperative prediction of LN metastasis in resectable PDAC using contrast-enhanced MRI. This offers a foundation for enhanced prognostic assessment and supports the management of personalized adjuvant treatment strategies.

**Key Points:**

- MRI-based habitat models can predict LN metastasis in PDAC.
- Both the radiomics model and clinical characteristics were useful for predicting LN metastasis in PDAC.
- The RHC models have the potential to enhance predictive accuracy and inform personalized therapeutic decisions.

**Graphical Abstract:**

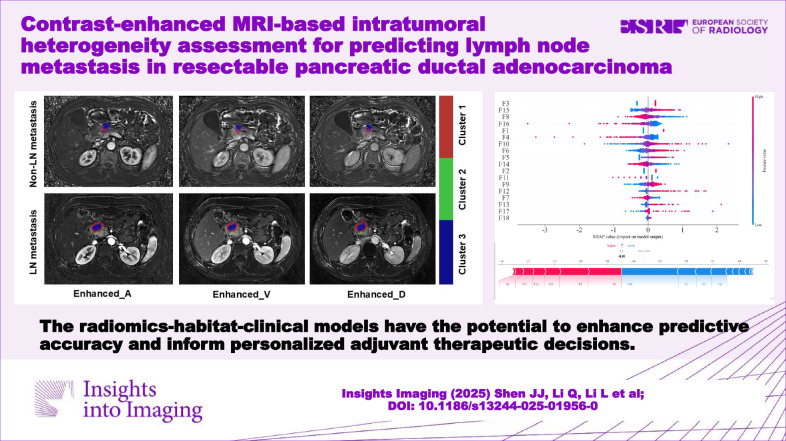

## Introduction

Pancreatic ductal adenocarcinoma (PDAC) is the third leading cause of cancer-related mortality, with a low 5-year survival rate of approximately 12% [1]. This poor prognosis is closely correlated with frequent LN metastasis [2, 3]. Notably, patients without LN metastasis have a 5-year survival rate four times higher than those with LN involvement, whose survival rate is only 10% [4].

The preoperative assessment of LN status is crucial for prognosis and treatment planning, as accurate assessment can guide neoadjuvant chemotherapy and improve outcomes for LN-positive PDAC patients [5–7]. Additionally, for patients with regional LN metastases, the benefit of surgical treatment may be reduced, representing the importance of timely and accurate detection of LN involvement [8]. The high heterogeneity of PDAC presents challenges for monitoring the biological behavior of the tumor and making clinical treatment decisions [9, 10]. This heterogeneity leads to variations in prognosis and immune response efficacy among patients with metastases to different sites, particularly among those with similar clinical features, such as lymph node (LN)-positive status [11]. Therefore, tumor biomarkers that reflect the overall characteristics of pancreatic cancer lesions but overlook ITH have limited value in assessing metastasis and prognosis.

Current noninvasive imaging techniques often struggle to differentiate LN metastasis from conditions like inflammation or lymphatic obstruction [12]. For instance, CT has a low sensitivity for detecting LN metastasis, around 25% [13]. Extracting radiological features from contrast-enhanced MRI holds promise for providing additional biologically relevant imaging information and has been shown to correlate with tumor diagnosis, treatment, and prognosis [14]. Radiomics can extract high-dimensional data from preoperative imaging to reveal subtle patterns often missed by traditional methods. Combining radiomics with advanced machine learning may enhance the accuracy of predicting LN metastasis in PDAC by distinguishing malignant nodes from benign ones.

Traditional radiomics, which extracts quantitative features from medical images, assumes uniform feature distribution within a lesion or its surrounding area. This approach does not account for ITH and may thus compromise prediction accuracy and reliability [15]. In contrast, habitat imaging, which segments tumors into distinct subregions or “habitats” based on specific structural and functional characteristics, provides a more detailed analysis of tumor heterogeneity [16, 17]. This method provides a detailed analysis of tumor heterogeneity by capturing regional variations in factors such as blood flow and cellular density, which conventional imaging techniques may overlook. Previous studies across various cancers have demonstrated that habitat imaging effectively assessed tumor biological behavior or prognosis, indicating that it could similarly refine LN involvement assessments [15, 18, 19]. Contrast-enhanced MRI, providing detailed non-invasive measurements of tumor biology and IHT, is well-suited for integration with habitat imaging. This combined approach enhances the visualization and monitoring of tumor heterogeneity through distinct sub-regions, potentially offering superior differentiation and evaluation of LN involvement [17].

Hence, in our large-sample, multicenter study, we aimed to develop and validate the value of the contrast-enhanced MRI-based intratumoral heterogeneity (ITH) model for predicting LN metastasis in resectable PDAC.

## Materials and methods

The retrospective study performed at Zhongshan Hospital, Fudan University, was approved by the institutional review board (B2024-250R) and was granted exemption from obtaining written informed consent. This protocol adhered to the ethical standards set forth by the 1964 Declaration of Helsinki and complied with the TRIPOD (Transparent Reporting of a Multivariable Prediction Model for Individual Prognosis or Diagnosis) guidelines.

### Patients selection

**Fig. 1:**
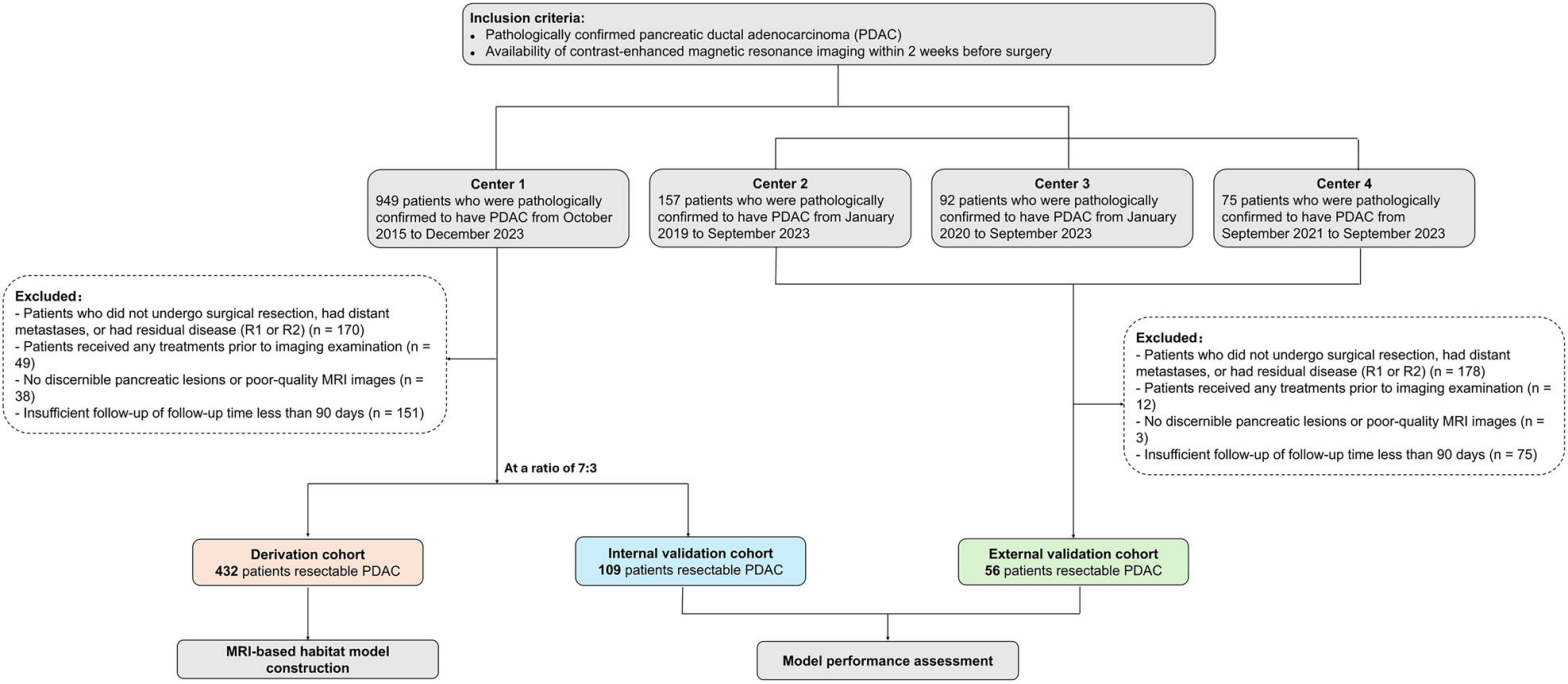
Flowchart of our study cohort

We collected data from consecutive patients who were treated for PDAC at a university teaching hospital (Zhongshan Hospital, Fudan University) between October 2015 and December 2023. In total, 949 patients with pathologically confirmed PDAC were reviewed in the study. In addition, an external validation cohort consisting of 324 patients with resectable PDAC was reviewed by enrolling participants from three different centers: 157 patients from center 2 between January 2019 and September 2023, 92 patients from center 3 between January 2020 and September 2023, and 75 patients from center 4 between September 2021 and September 2023. The criteria for inclusion and exclusion are shown in Fig. 1. A total of 597 patients with PDAC were finally enrolled, including 432 in the derivation cohort, 109 in the internal validation cohort, and 56 in the external validation cohort.

### MRI protocol

Due to the extended duration and multicenter nature of the study, MRI scans were acquired using various 1.5-T or 3.0-T MRI systems. Comprehensive details regarding the MRI protocols can be found in Appendix S1, and Tables E1 and E2.

### Clinical and radiological characteristics

The study assessed a range of demographic and laboratory variables, including age, sex, the presence of diabetes and hypertension, serum levels of cancer antigen 19-9 (CA 19-9), total bilirubin (TBil), and albumin.

As respect to radiological characteristics, two abdominal radiologists, with 10 years and 23 years of experience in abdominal MRI respectively, performed a retrospective review of the imaging data, remaining unaware of any additional clinical and pathological details. Each radiologist assessed the imaging characteristics of the PDAC cases independently. Discrepancies in their evaluations were addressed by a third senior radiologist with 31 years of expertise in pancreatic MRI, who reviewed the images to achieve a final consensus. Tumors were evaluated for the following features: (a) tumor size, defined as the maximum cross-sectional diameter; (b) location, categorized as either the pancreatic head/neck or the body/tail; and (c) radiological-enlarged LN as determined by MRI imaging if a short-axis diameter of the LN exceeded 10 mm.

### Pathologic feature analysis

The pathological data were extracted from the patient’s medical records. A pathologist with 15 years of experience in abdominal pathology re-evaluated the pT and pN stages. Detailed pathological findings were documented, including: (a) extent of invasion (pT) and LN involvement (pN) based on the eighth edition of the American Joint Committee on Cancer (AJCC) staging system, (b) tumor differentiation grade, (c) pathological lymphovascular invasion (PLI), (d) perineural invasion (PNI), and (e) pathological fatty infiltration (PFI).

### Image segmentation and image preprocessing

All images were manually annotated across three contrast-enhanced sequences: T1-weighted images at the arterial phase (T1_A), T1-weighted images at the venous phase (T1_V), and T1-weighted images at the delayed phase (T1_D). These annotations were then used for subsequent analysis. The detailed process is listed in Appendix S2.

### Habitat imaging and radiomic feature extraction

After registration, each voxel in the tumor region integrated the value from the calculated parametric mappings to form a three-dimensional vector. To prevent features with high numerical values from dominating the clustering process, *z*-score normalization was applied to ensure the mappings were normally distributed. Within the feature space, the *K*-means algorithm was used to conduct the habitat imaging. The detailed process is listed in Appendix S3.

### Feature selection, habitat model construction, and evaluation

Three algorithms were applied sequentially for feature selection. First, SelectKBest was used to select the top 100 features correlated with the label via an *F*-test. Second, the selected 100 features were processed using mRMR (Maximal Relevance and Minimal Redundancy) to identify a subset of 50 features with maximum correlation and minimal redundancy, based on information theory. Finally, LASSO (Least Absolute Shrinkage and Selection Operator) was employed to select the most valuable features. Three enhancement ratio maps—Enhanced_A (arterial phase), Enhanced_V (venous phase), and Enhanced_D (delayed phase)—were generated by subtracting pre-contrast images from their contrast-enhanced counterparts, and the Enhanced_habitat model was constructed using these maps. All other model construction and evaluation were outlined in detail in Appendix S4.

### Statistical analysis

**Fig. 2:**
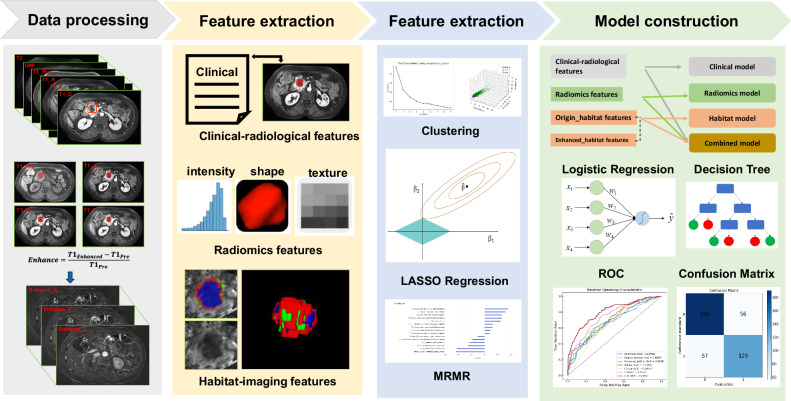
The technical outline for this study

Statistical analysis was conducted using SPSS software (version 26.0, IBM) and R language (version 4.4.1). Continuous variables were compared using the student’s *t*-test or Mann–Whitney *U*-test, while categorical variables were analyzed using the Chi-square test or Fisher’s exact test, as applicable. The DeLong test was employed to compare the area under the curve (AUC) of the various models. The optimal cutoff value (0.46) was determined via radiomics–habitat–clinical (RHC)-predicted score categorizing patients into high-risk or low-risk groups. The prognostic value of various parameters or models for prognosis was evaluated using Kaplan–Meier survival analysis with the log-rank test. A two-tailed *p*-value < 0.05 was considered statistically significant. The whole workflow is presented in Fig. 2.

## Results

### Patient characteristics

Five hundred ninety-seven patients with PDAC across the three cohorts were enrolled in this study. The baseline clinical, radiological, and pathological characteristics are summarized in Table 1. In our center’s derivation and internal validation cohorts, the overall incidence of LN metastasis was 43.1% (233 of 541 cases), with pN2 accounting for 6.1% (33 of 541 cases). In the external validation cohort, the overall incidence of LN metastasis was 39.3% (22 of 56 cases), with pN2 accounting for 16.1% (9 of 56 cases). LN metastasis was observed in 186 (43.0%), 47 (43.1%), and 22 (39.3%) patients in the training, internal validation, and external validation sets, respectively. In the training set, CA19-9, TBil, and Radiological-enlarged LN status were retained after conducting both univariate and multivariate logistic regression analyses (Table 2), LN metastasis was more frequently associated with elevated CA19-9 level (OR: 1.248; 95% CI: 1.020–1.527) and increased TBil level (OR: 1.243; 95% CI: 1.019–1.517) and the presence of radiologically-enlarged LN (OR: 1.320; 95% CI: 1.086–1.605). The variables were subsequently integrated into the clinical model, yielding an AUC of 0.641 (95% CI: 0.590–0.693) in the derivation cohort, 0.672 (95% CI: 0.571–0.774) in the internal validation cohort, and 0.615 (95% CI: 0.465–0.766) in the external validation cohort, respectively.

**Table 1:** Baseline clinical, radiological, and pathological characteristics

Parameters	Derivation (*n* = 432)		Internal validation (*n* = 109)		*p* value	External validation (*n* = 56)	
	Non-LN metastasis (*n* = 246)	LN metastasis (*n* = 186)	Non-LN metastasis (*n* = 62)	LN metastasis (*n* = 47)		Non-LN metastasis (*n* = 34)	LN metastasis (*n* = 22)
Age (years)^*^	67.0 [60.0, 72.0]	66.0 [60.0, 70.0]	66.0 [57.3, 72.0]	65.0 [59.5, 73.0]	0.923	65.0 [59.3, 68.8]	67.0 [60.0, 70.8]
Sex					0.489		
Female	112 (45.5)	75 (40.3)	28 (45.2)	15 (31.9)		18 (52.9)	6 (27.3)
Male	134 (54.5)	111 (59.7)	34 (54.8)	32 (68.1)		16 (47.1)	16 (72.7)
Diabetes					0.140		
Absence	176 (71.545)	129 (69.355)	40 (64.516)	29 (61.702)		30 (88.2)	14 (63.6)
Presence	70 (28.455)	57 (30.645)	22 (35.484)	18 (38.298)		4 (11.8)	8 (36.4)
Hypertension					0.111		
Absence	138 (56.1)	107 (57.5)	42 (67.7)	29 (61.7)		27 (79.4)	17 (77.3)
Presence	108 (43.9)	79 (42.5)	20 (32.3)	18 (38.3)		7 (20.6)	5 (22.7)
TBil (μmol/L)					0.986		
< 22.2	175 (71.1)	106 (57.0)	44 (71.0)	27 (57.4)		26 (76.5)	11 (50.0)
≥ 22.2	71 (28.9)	80 (43.0)	18 (29.0)	20 (42.6)		8 (23.5)	11 (50.0)
Albumin (g/L)					0.634		
< 35	219 (89.0)	172 (92.5)	55 (88.7)	42 (89.4)		32 (94.1)	21 (85.5)
≥ 35	27 (11.0)	14 (7.5)	7 (11.3)	5 (10.6)		2 (5.8)	1 (4.5)
CA19-9 (U/mL)					0.402		
< 100	117 (47.6)	62 (33.3)	32 (51.6)	18 (38.3)		12 (35.3)	10 (45.5)
≥ 100	129 (52.4)	124 (66.7)	30 (48.4)	29 (61.7)		22 (64.7)	12 (54.5)
Size (cm)^*^	2.5 [2.0, 3.5]	3.0 [2.2, 3.5]	2.2 [2.0, 3.5]	3.0 [2.5, 4.0]	0.963	3.3 [2.6, 4.0]	2.9 [2.0, 3.5]
Location					0.674		
Head	146 (59.4)	118 (63.4)	38 (61.3)	31 (66.0)		23 (67.6)	18 (81.8)
Body/tail	100 (40.6)	68 (36.6)	24 (38.7)	16 (34.0)		11 (32.4)	4 (18.2)
Radiological-enlarged LN					0.264		
Absence	205 (83.3)	130 (69.9)	52 (83.9)	27 (57.4)		30 (88.2)	15 (68.2)
Presence	41 (16.7)	56 (30.1)	10 (16.1)	20 (42.6)		4 (11.8)	7 (31.8)
AJCC 8th pT stage					0.941		
T1	75 (30.4)	39 (20.9)	24 (38.7)	6 (12.8)		5 (14.7)	3 (13.6)
T2	132 (53.7)	124 (66.7)	32 (51.6)	31 (66.0)		19 (55.9)	15 (68.2)
T3	39 (15.9)	23 (12.4)	6 (9.7)	10 (21.3)		10 (29.4)	4 (18.2)
AJCC 8th staging					0.851		
I	199 (80.9)	0 (0.0)	53 (85.5)	0 (0.0)		22 (64.7)	0 (0.0)
II	36 (14.6)	151 (81.2)	4 (6.4)	40 (85.1)		11 (32.4)	20 (90.9)
III	11 (4.5)	35 (18.8)	5 (8.1)	7 (14.9)		1 (2.9)	2 (9.1)
Differentiation grade					0.364		
Well/moderately	145 (58.9)	90 (48.4)	33 (53.2)	21 (44.7)		19 (55.9)	14 (63.6)
Poor	101 (41.1)	96 (51.6)	29 (46.8)	26 (55.3)		15 (44.1)	8 (36.4)
PNI					0.757		
Absence	43 (17.5)	27 (14.5)	14 (22.6)	5 (10.6)		3 (8.8)	2 (9.1)
Presence	203 (82.5)	159 (85.5)	48 (77.4)	42 (89.4)		31 (91.2)	20 (90.9)
PLI					0.149		
Absence	194 (78.9)	113 (60.8)	56 (90.3)	29 (61.7)		26 (76.5)	12 (54.5)
Presence	52 (21.1)	73 (39.2)	6 (9.7)	18 (38.3)		8 (23.5)	10 (45.5)
PFI					0.120		
Absence	57 (23.2)	25 (13.4)	21 (33.2)	7 (14.9)		8 (23.5)	6 (27.3)
Presence	189 (76.8)	161 (86.6)	41 (66.1)	40 (85.1)		26 (76.5)	16 (72.7)
RFS					0.464		
Non-recurrence	126 (51.2)	79 (42.5)	36 (58.0)	20 (42.6)		16 (47.1)	14 (63.6)
Recurrence	120 (48.8)	107 (57.5)	26 (42.0)	27 (57.4)		18 (52.9)	8 (36.4)
OS					0.732		
Alive	183 (74.4)	131 (70.4)	54 (87.1)	27 (57.4)		28 (82.4)	16 (72.7)
Dead	63 (25.6)	55 (29.6)	8 (12.9)	20 (42.6)		6 (17.6)	6 (27.3)
RFS time (day)^*^	357 [168, 685]	276 [128, 527]	311 [162, 648]	292 [163, 485]		290 [136, 469]	440 [173, 803]
OS time (day)^*^	512 [258, 1013]	441 [203, 837]	384.5 [245, 825]	431.0 [198, 785]		468 [192, 745]	473 [179, 896]

*p* value was measured via Pearson’s Chi-square test (two-sided) comparing derivation and internal validation cohorts

*TBil* total bilirubin, *CA 19-9* cancer antigen 19-9, *AJCC* American Joint Committee on Cancer, *PNI* perineural invasion, *PLI* pathological lymphovascular invasion, *PFI* pathological fatty infiltration, *RFS* recurrence-free survival, *OS* overall survival

* Data are median (interquartile ranges), other parameters are expressed as *n* (%)

**Table 2:** Univariate and multivariate regression analysis of LN metastasis

Parameters	Univariate analysis					Multivariate analysis				
	Coefficient	OR	95CI_L	95CI_U	*p* value	Coefficient	OR	95CI_L	95CI_U	*p* value
Age (years)	−0.074	0.929	0.768	1.124	0.447					
Sex (male)	0.105	1.111	0.918	1.345	0.280					
Diabetes (presence)	0.048	1.049	0.868	1.269	0.621					
Hypertension (presence)	−0.029	0.972	0.803	1.176	0.767					
TBil (> 100 μmol/L)	0.296	1.344	1.111	1.627	**0.002**	0.217	1.243	1.019	1.517	**0.032**
Albumin (< 35 g/L)	−0.061	0.940	0.777	1.138	0.528					
CA19-9 (> 100 U/mL)	0.293	1.341	1.104	1.628	**0.003**	0.221	1.248	1.020	1.527	**0.032**
Location (Head)	−0.084	0.919	0.759	1.113	0.388					
Tumor size (cm)	0.016	1.017	0.841	1.229	0.866					
Radiological- enlarged LN (presence)	0.320	1.377	1.137	1.668	**0.001**	0.278	1.320	1.086	1.605	**0.005**

The bold values indicate statistical significance

*TBil* total bilirubin, *CA 19-9* cancer antigen 19-9

### ITH analysis and radiomic feature selection

**Fig. 3:**
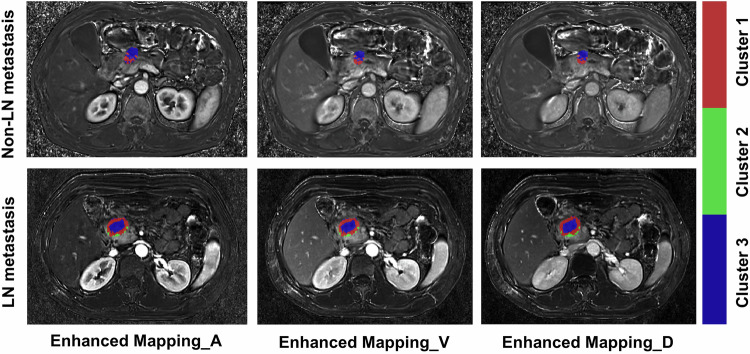
Representative MRI images of patients with LN-positive and LN-negative metastasis in Enhanced Ratio Mapping_arterial (Enhanced_A), Enhanced Ratio Mapping_venous (Enhanced_V), and Enhanced Ratio Mapping_delayed (Enhanced_D) sequences. Each color displays a distinct tumor habitat

Based on the Calinski–Harabasz score, the ideal number of clusters for the tumor region of interest is identified as 3, resulting in the identification of three distinct subregions in the derivation cohort (Fig. E1). For the subregion proportion, habitat 2 was the most predominant (46.4%), followed by habitats 3 (37.3%) and 1 (16.3%). A total of 2264 radiomics features and 1904 habitat features were extracted for both radiomics and habitat analyses. Following feature reduction and LASSO selection, the Radiomics model, Origin habitat, Enhanced habitat, Habitat, and RH model retained 8, 10, 6, 13, and 15 features, respectively (Fig. E2 and Table E3–4). Moreover, the hierarchical clustering heatmap suggested the radiomics and various habitat models-selected features were effective in distinguishing LN metastasis (Fig. E3). The two most significant features were derived from the Radiomics model and the Enhanced_D habitat model. Additionally, Fig. 3 presents representative habitat imaging from both the LN metastasis and non-LN metastasis groups.

## Combination models and performance evaluation

### Radiomics models from each single sequence

The radiomics models based on six individual sequences—Radiomic_DWI, Radiomic_T2, Radiomic_T1_Pre, Radiomic_T1_A, Radiomic_T1_V, and Radiomic_T1_D—achieved AUCs ranging from 0.613 to 0.681 in the derivation cohort and 0.600–0.674 in the internal validation cohort for predicting LN metastasis. When these models were combined into an ensemble radiomics model using the LR method, the AUCs were 0.679 (95% CI: 0.628–0.729), 0.675 (95% CI: 0.573–0.778), and 0.574 (95% CI: 0.402–0.746) for the derivation, internal validation, and external validation cohorts, respectively. Detailed performance metrics for each radiomics model based on individual sequences are provided in Table E5.

### Habitat-based models from enhanced mapping and single sequences

We classified all tumor pixels into three groups using the *K*-means clustering algorithm, denoted as Cluster1, Cluster2, and Cluster3. For each sequence of the tumor in all patients, models corresponding to all three regions were subsequently generated (e.g., Habitat_Enhanced_A_1, Habitat_Enhanced_A_2, Habitat_Enhanced_A_3). In predicting LN metastasis, the habitat models derived from the following seven sequences—T1_Pre, T1_A, T1_V, T1_D, Enhanced_A, Enhanced_V, and Enhanced_D—achieved AUCs ranging from 0.548 to 0.690 in the derivation cohort and from 0.424 to 0.653 in the internal validation cohort. (Table E6). Table E6 provides a comprehensive overview of predictive metrics, including sensitivity, specificity, accuracy, precision, and F1 score.

The Original_habitat model (combining the T1_Pre, T1_A, T1_V, and T1_D sequences), the Enhanced_habitat model (combining Enhanced_A, Enhanced_V, and Enhanced_D), and the ensembled habitat model (integrating both Original_habitat and Enhanced_habitat), demonstrated AUCs of 0.684 (95% CI: 0.634–0.734), 0.663 (95% CI: 0.611–0.715), and 0.716 (95% CI: 0.668–0.764) in the derivation cohort; 0.665 (95% CI: 0.559–0.772), 0.653 (95% CI: 0.550–0.756), and 0.706 (95% CI: 0.605–0.808) in the internal validation cohort; and 0.689 (95% CI: 0.537–0.842), 0.667 (95% CI: 0.523–0.811), and 0.708 (95% CI: 0.567–0.848) in the external validation cohort, respectively (Table 3).

**Table 3:** Performance of various models for predicting LN metastasis

Model	AUC (95% CI)	ACC	SEN	SPEC	Precision	F1-score
Deviation						
Radiomics	0.679 (0.628–0.729)	0.644	0.392	0.833	0.640	0.487
Original_habitat	0.684 (0.634–0.734)	0.606	0.683	0.549	0.534	0.599
Enhanced_habitat	0.663 (0.611–0.715)	0.625	0.651	0.606	0.555	0.599
Habitat model	0.716 (0.668–0.764)	0.657	0.559	0.732	0.612	0.584
RH	0.752 (0.707–0.798)	0.634	0.828	0.488	0.550	0.661
Clinical	0.641 (0.590–0.693)	0.630	0.532	0.703	0.576	0.553
RHC	0.805 (0.763–0.847)	0.745	0.677	0.797	0.716	0.696
Internal validation						
Radiomics	0.675 (0.573–0.778)	0.688	0.468	0.855	0.710	0.564
Original_habitat	0.665 (0.559–0.772)	0.670	0.702	0.645	0.600	0.647
Enhanced_habitat	0.653 (0.55–0.756)	0.624	0.617	0.629	0.558	0.586
Habitat model	0.706 (0.605–0.808)	0.679	0.617	0.726	0.630	0.624
RH	0.729 (0.631–0.827)	0.688	0.851	0.565	0.597	0.702
Clinical	0.672 (0.571–0.774)	0.679	0.660	0.694	0.620	0.639
RHC	0.779 (0.688–0.870)	0.761	0.723	0.790	0.723	0.723
External validation						
Radiomics	0.574 (0.402–0.746)	0.589	0.609	0.576	0.500	0.549
Original_habitat	0.689 (0.537–0.842)	0.679	0.478	0.818	0.647	0.550
Enhanced_habitat	0.667 (0.523–0.811)	0.607	0.087	0.970	0.667	0.154
Habitat model	0.708 (0.567–0.848)	0.643	0.261	0.909	0.667	0.375
RH	0.655 (0.508–0.802)	0.643	0.435	0.788	0.588	0.500
Clinical	0.615 (0.465–0.766)	0.625	0.391	0.788	0.562	0.462
RHC	0.734 (0.595–0.873)	0.732	0.565	0.848	0.722	0.634

### Ensembled models and performance analysis

**Fig. 4:**
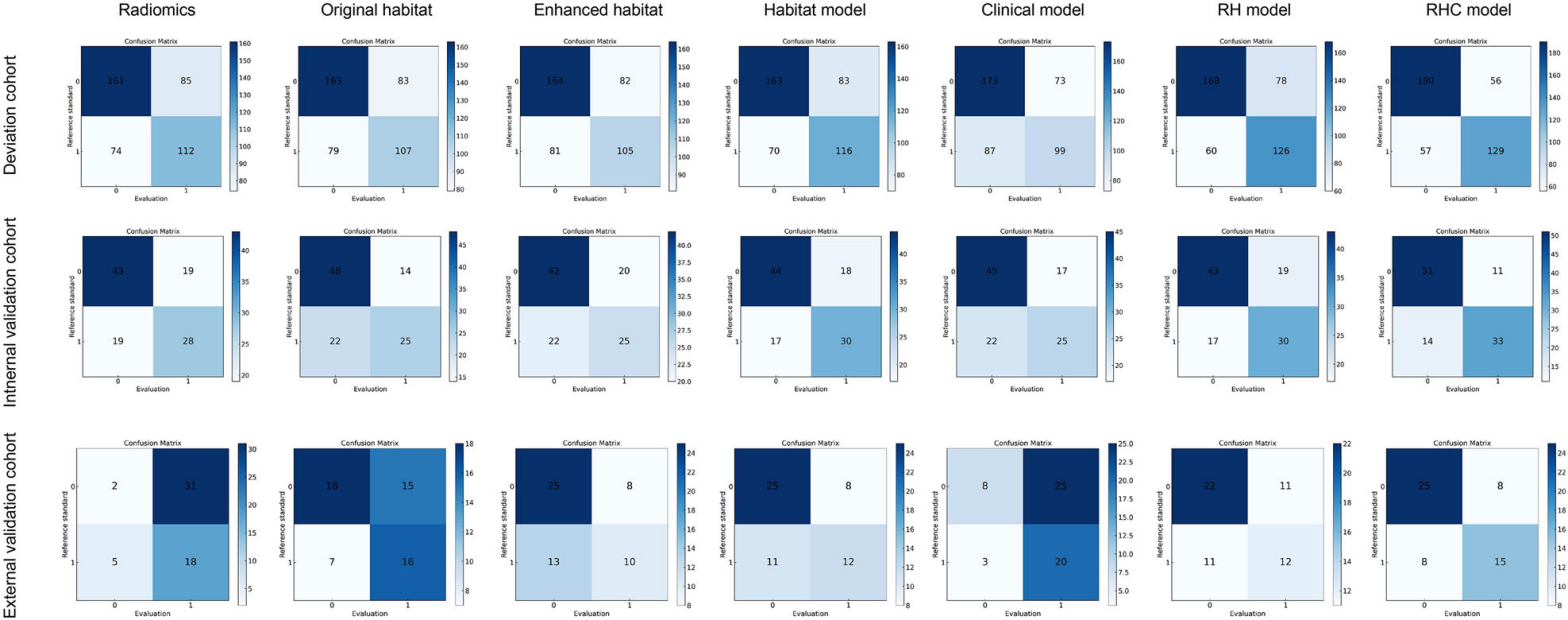
Confusion matrix of seven models in the deviation, internal validation, and external cohort

**Fig. 5:**
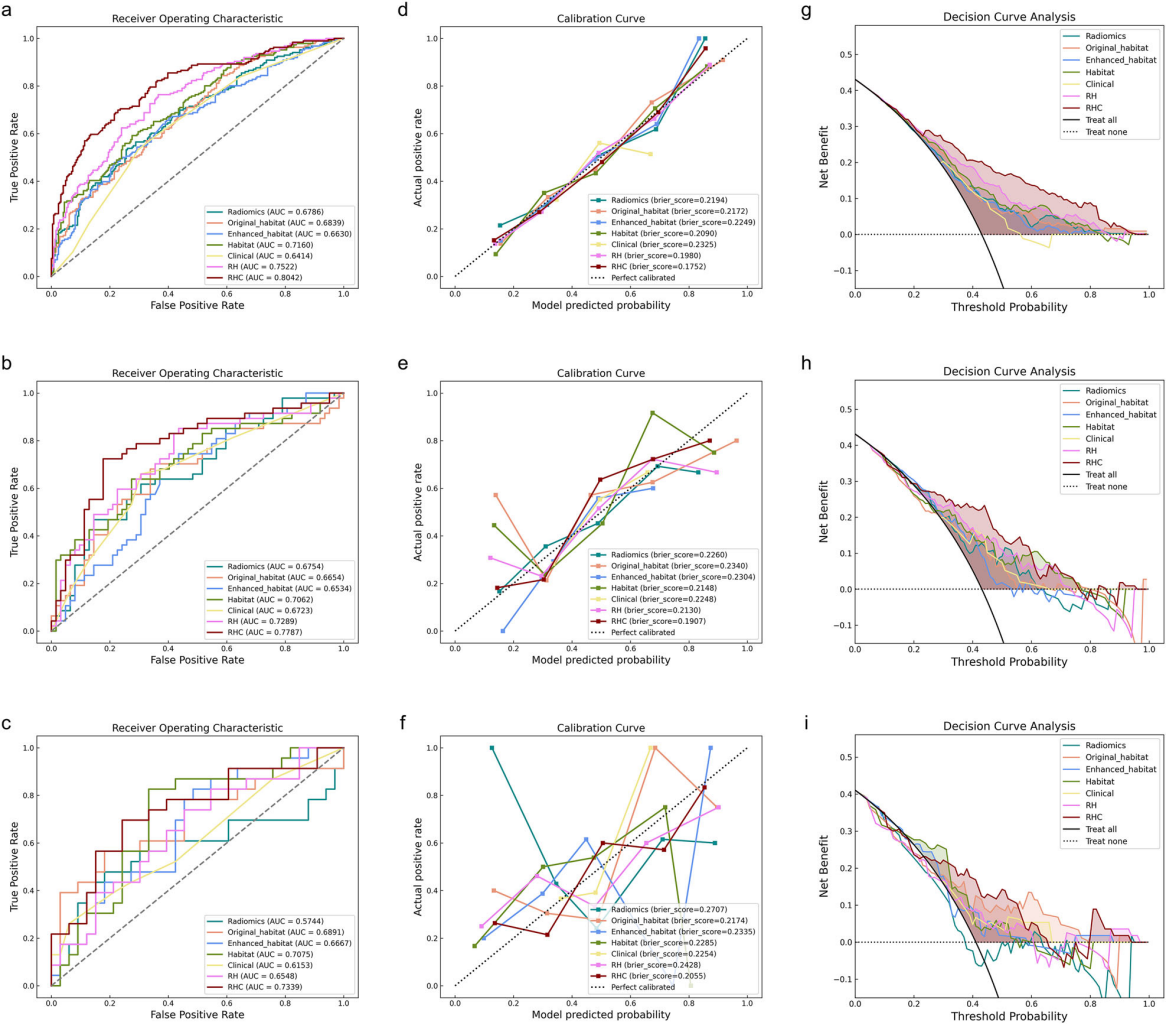
Capacity of various models for predicting LN metastasis. **a** Receiver operating characteristic curves, calibration curves, and decision curves of seven models in the deviation (**a**–**c**), internal validation (**d**–**f**), and external validation (**g**–**i**) cohorts

For the imaging-based prediction of LN metastasis, the RH model, which integrates Radiomics and Habitat features using the LR method and selects fifteen features, achieved an AUC of 0.752 (95% CI: 0.707–0.798) in the derivation cohort, 0.729 (95% CI: 0.631–0.827) in the internal validation cohort, and 0.655 (95% CI: 0.508–0.802) in the external validation cohort. These results are superior to those obtained from the individual Radiomics, Original_habitat, and Enhanced_habitat models. Additionally, the ensembled RHC, which integrates Radiomics, Habitat, and Clinical features using the SVM method, achieved the highest AUC values of 0.805 (95% CI: 0.763–0.847), 0.779 (95% CI: 0.688–0.870), and 0.615 (95% CI: 0.465–0.766) in the derivation, internal validation, and external validation cohorts, respectively (Table 3). Moreover, Table 3 summarizes the various additional predictive metrics of different ensembled models. The confusion matrices of the seven models for binary classification in predicting LN metastasis are shown in Fig. 4. AUC curves, calibration curves, and decision curve analysis (DCA) demonstrated that the RHC model exhibited superior predictive performance in distinguishing LN status compared to the other models (Fig. 5 and Table E7).

The SHapley Additive exPlanations (SHAP) algorithm was utilized to produce an importance ranking, providing interpretability for the output of the final ensembled RHC model by calculating SHAP values for the 18 selected features (Fig. E4 and Table E8).

### Predictive value of the ensembled model for prognosis risk stratification and adjuvant therapy outcomes

**Fig. 6:**
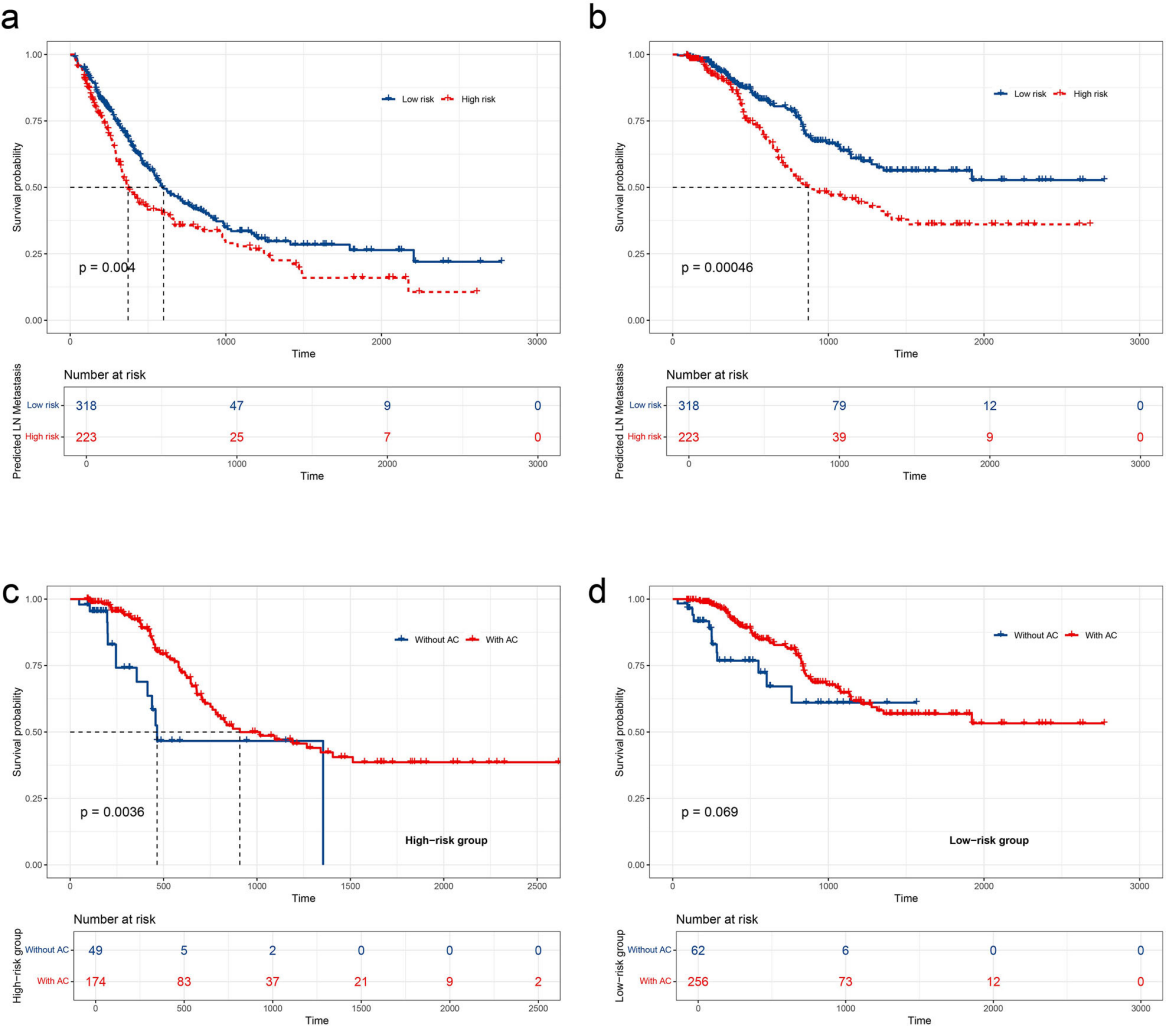
Kaplan-Meier (KM) survival curves of recurrence-free (**a**) and OS (**b**) according to the final RHC model in the entire cohort (center 1). Additionally, KM survival curves of OS outcomes in patients at (**c**) high- and (**d**) low-risk groups for patients with or without adjuvant therapies (AC)

Based on the cut-off values derived from the ensembled RHC model-predicted LN metastasis in the entire cohort (center 1), patients were categorized into low-risk and high-risk groups. Kaplan-Meier survival curves showed that the final RHC model can provide the additional value of distinguishing recurrence-free survival (RFS) and OS outcomes for patients with resectable PDAC (median RFS, 600 vs 331 days; *p* = 0.004; median OS, 871 vs *NA* days; *p* < 0.001) (Fig. 6a, b).

Additionally, adjuvant therapies demonstrated improved survival outcomes in the RHC model-predicted high-risk group for LN metastasis, with a significantly longer median OS (median OS, 908 vs 465 days; *p* = 0.004). However, no statistically significant survival benefit was observed in the model-predicted low-risk group for LN metastasis (*p* = 0.069) (Fig. 6c, d).

## Discussion

In this study, we utilized a multiparametric MRI-based ITH subregion to develop an ensembled RHC model for predicting the LN metastasis for resectable PDAC. In addition, we applied an RHC-derived score for LN metastasis risk stratification, effectively distinguishing OS and RFS risks while offering valuable guidance for optimizing adjuvant treatment strategies.

LN metastasis in PDAC is closely associated with surgical planning and patient prognosis [20–22]. However, conventional imaging modalities, such as CT and MRI, rely on LN size, morphology, and signal/density characteristics for evaluation, which are subject to the limitations of radiologists’ subjective interpretations, resulting in sufficient sensitivity and suboptimal diagnostic performance [23]. Additionally, several clinical factors, such as serum CA19-9, tumor size, etc., have been reported to correlate with LN metastasis [24–26]. In our study, multivariate regression analysis identified CA19-9 levels (> 100 U/mL, OR: 1.248; 95% CI: 1.020–1.527), TBil levels (> 22.2 μmol/L, OR: 1.243; 95% CI: 1.019–1.517), and radiologically enlarged LNs (OR: 1.320; 95% CI: 1.086–1.605) as independent risk factors for LN metastasis in resectable PDAC. However, the predictive performance of the clinical model based on these factors was modest, with AUCs of 0.672 and 0.655 in the internal and external validation cohorts, respectively.

Currently, radiomics-based imaging models are progressively being employed to predict LN metastasis in patients with PDAC [24, 27, 28]. A recent meta-analysis indicates that radiomics models can achieve relatively superior sensitivity and specificity compared to conventional semantic interpretations of radiological images [29]. However, studies focusing on MRI-based radiomics for predicting LN metastasis in PDAC patients remain limited. Zhenshan Shi et al developed an MRI radiomics model, achieving AUC values of 0.868 and 0.772 for the training and validation cohorts, respectively [30]. Additionally, research by Lin Shi et al reported an MRI-based radiomics nomogram model for predicting LN metastasis, with AUC values of 0.845 and 0.816 in the primary and validation cohorts, respectively [31]. Nonetheless, it is crucial to note that the primary limitation of these studies is model overfitting, further complicated by small or single-center sample sizes, which can lead to overly optimistic AUC values and poor reproducibility. In our study, the performance of a standalone radiomics model yielded AUC values of 0.679 and 0.675 in the training and internal validation cohorts, respectively; however, its efficacy in the external validation cohort was suboptimal, with an AUC of only 0.574 and a relatively high Brier score of 0.270. Therefore, although radiomics can extract and identify some effective features, a single radiomics model may struggle to meet the clinical demands for accurately predicting LN metastasis in resectable patients.

Habitat analysis enables the segmentation of tumors and the identification of corresponding habitat characteristics that reflect tumor heterogeneity [32]. Consequently, habitat imaging can serve as an indicator of ITH, where an increase in the number of habitat subregions correlates with greater tumor heterogeneity [33, 34]. The pronounced heterogeneity of tumors is a hallmark feature of PDAC, and the heterogeneity within and between PDAC tumors plays a critical role in treatment resistance and prognosis. A recent study by Esther M.M. Smeets demonstrated that [^18^F]FDG PET-based subregions are associated with high/heterogeneous MCT4 subgroups, MCT4 expression levels, and local variations in pancreatic cancer. This suggests that subregion-based radiomics scores have a strong potential for non-invasively capturing intratumoral marker heterogeneity and identifying subgroups of PDAC patients with poor prognoses [35].

The signal enhancement ratio (SER) in multi-phase contrast-enhanced MR imaging is closely correlated with PDAC tissue components and prognosis [36]. Hence, in our study, we performed subtraction processing on MR images to generate arterial, venous, and delayed enhancement ratio images for subregion clustering analysis. This approach not only facilitated the identification of features related to ITH but also transformed the originally non-quantitative MR images into quantitatively analyzable subtraction images, which helped to reduce the variability introduced by different MR machines. As expected, among the enhanced_habitat, habitat, and RH models, the enhanced_D mapping-derived habitat features (binomialblurimage_ngtdm_Contrast) proved to be the most significant in distinguishing LN metastasis of PDAC. As for results, the habitat and radiomics-habitat (RH) model showed the AUCs of 0.706, 0.708, and 0.729, 0.655 in the internal and external validation cohorts, respectively, better than traditional clinical and radiomics models. On this basis, imaging and clinical models were further integrated to obtain a better and more practical LN metastasis prediction model for PDAC. The integrated RHC model demonstrated the best predictive performance across the training set, internal validation set, and external validation set, achieving the highest AUC values of 0.804, 0.779, and 0.734, respectively, along with the lowest Brier scores of 0.175, 0.191, and 0.205. According to the RHC model-derived risk stratification, the high-risk group exhibited shorter median RFS and overall survival (OS) times. More notably, in the subgroup analysis, patients in the high-risk group who received postoperative adjuvant treatment demonstrated a longer median OS time (908 vs 465 days). However, there were no prognostic differences observed among patients in the low-risk group. This indicates that the contracted model not only serves as a non-invasive preoperative predictor of LN metastasis but also offers valuable insights for prognostic risk assessment and the formulation of personalized adjuvant treatment strategies for candidates with resectable PDAC.

Our study still has some limitations. Firstly, although this is a multicenter study, the sample size of the external validation group remains relatively small. Further efforts are needed to expand the number of participating centers and increase the sample size for more comprehensive model validation. Additionally, the heterogeneity in MRI protocols in our retrospective study, including variations in machines, contrast administration, and acquisition parameters (e.g., arterial phase flow rates), may have introduced variability in the results, potentially affecting their generalizability. Due to technical challenges and ethical considerations, the spatially distinct habitats identified through *K*-means clustering have not been validated pathologically. To further confirm the biological significance of our habitat subregions, subsequent studies should involve matching and sampling tissue specimens from surgical patients with the MRI subregions for bioinformatics analysis and validation.

In conclusion, MRI-based ITH assessment holds the potential to predict LN metastasis in PDAC. Additionally, the RHC model exhibited the highest predictive accuracy for LN metastasis in resectable PDAC patients, providing additional value for prognostic assessment and aiding in the development of personalized adjuvant treatment strategies.

## Supplementary information

ELECTRONIC SUPPLEMENTARY MATERIAL

## Data Availability

The datasets used and/or analyzed during the current study are available from the corresponding author upon reasonable request.

## Abbreviations

AJCC: American Joint Committee on Cancer
AUC: Area under the curve
CA 19-9: Cancer antigen 19-9
ITH: Intratumoral heterogeneity
LN: Lymph node
OS: Overall survival
PDAC: Pancreatic ductal adenocarcinoma
PFI: Pathological fatty infiltration
PLI: Pathological lymphovascular invasion
PNI: Perineural invasion
RFS: Recurrence-free survival
TBil: Total bilirubin
