# Correction: Contrast-enhanced MRI-based intratumoral heterogeneity assessment for predicting lymph node metastasis in resectable pancreatic ductal adenocarcinoma

**DOI:** 10.1186/s13244-025-01973-z

**Published:** 2025-05-15

**Authors:** Junjian Shen, Qing Li, Lei Li, Tianyu Lu, Jun Han, Zongyu Xie, Peng Wang, Zirui Cao, Mengsu Zeng, Jianjun Zhou, Tianzhu Yu, Yaolin Xu, Haitao Sun

**Affiliations:** 1 Department of Radiology, Zhongshan Hospital, Fudan University, Shanghai Institute of Medical Imaging, Shanghai, China; 2 Department of Radiology, Fengyang County People’s Hospital, Chuzhou, China; 3 Department of Radiology, The First Hospital of Jiaxing, Affiliated Hospital of Jiaxing University, Jiaxing, China; 4 Department of Radiology, The First Affiliated Hospital of Bengbu Medical University, Bengbu, China; 5 Department of Radiology, Affiliated Hospital of Jiangnan University, Wuxi, P.R. China; 6 Department of Research Center, Shanghai United Imaging Intelligence Co., Ltd., Shanghai, China; 7 Department of Radiology, Zhongshan Hospital (Xiamen), Fudan University, Xiamen Municipal Clinical Research Center for Medical Imaging, Fujian Province Key Clinical Specialty for Medical Imaging, Xiamen Key Laboratory of Clinical Transformation of Imaging Big Data and Artificial Intelligence, Xiamen, China; 8 Department of Interventional Radiolog9loy, Zhongshan Hospital, Shanghai, China; 9 Department of Pancreatic Surgery, Zhongshan Hospital, Fudan University, Shanghai, China

**Correction to:** **Insights into Imaging** (2025) 16:76;

10.1186/s13244-025-01956-0; published online 30 March 2025

In this article the author’s name Li Qing was incorrectly written as Li Qin.

The original article has been corrected.

