# Supplementary material for: Contrast-enhanced MRI-based intratumoral heterogeneity assessment for predicting lymph node metastasis in resectable pancreatic ductal adenocarcinoma
Source: Insights Imaging. 2025 Mar 30;16:76. doi: 10.1186/s13244-025-01956-0 (PMC11955437; doi:10.1186/s13244-025-01956-0)
Supplement: Supplementary file 1 — ELECTRONIC SUPPLEMENTARY MATERIAL [file 13244_2025_1956_MOESM1_ESM.pdf]

Contrast-enhanced MRI-Based Intratumoral Heterogeneity Assessment for Predicting Lymph Node Metastasis in Resectable Pancreatic Ductal Adenocarcinoma

ELECTRONIC SUPPLEMENTARY MATERIAL

Appendix S1

Detailed Imaging Acquisition

All contrast-enhanced MRIs were performed using the following MRI systems: [MAGNETOM Aera, Siemens; Prisma, Siemens], [GE 3.0T Discovery MR750, Siemens Aera], [GE MEDICAL SYSTEMS SIGNA Architect, Philips Ingenia CX], and [Philips Ingenia CX 3.0T]. Gadolinium-based contrast agents, including gadolinium diethylenetriamine pentaacetic acid (Gd-DTPA; Magnevist, Bayer HealthCare), gadobenate dimeglumine (Xudong Haipu Pharmaceutical, Shanghai, China), and gadobenate dimeglumine (Beilu Pharmaceutical, Beijing, China), were intravenously injected and reached the ascending aorta. The Gd-DTPA agents were administered at a dosage of 0.1 mmol/kg, with an infusion rate of 1.0–2.0 mL/s for gadolinium diethylenetriamine pentaacetic acid (Magnevist, Bayer HealthCare), gadobenate dimeglumine (Xudong Haipu Pharmaceutical, Shanghai, China), or gadobenate dimeglumine (Beilu Pharmaceutical, Beijing, China).

Prior to MRI scans, patients were required to fast for 6–8 hours. The MRI protocol consisted of the following sequences: (a) T2-weighted imaging; (b) diffusion-weighted imaging with b values set to 0, 50, and 500 s/mm² for [MAGNETOM Aera, Siemens; Prisma, Siemens] systems, 0, 50, and 800 s/mm² for [GE MEDICAL SYSTEMS SIGNA Architect], or 0 and 800 s/mm² for [Philips Ingenia CX 3.0T; GE 3.0T Discovery MR750, Siemens Aera]. Apparent diffusion coefficient (ADC) maps were then generated using a monoexponential model. Additionally, (c) in-phase and out-of-phase T1-weighted imaging, and (d)

dynamic T1-weighted imaging were performed during the precontrast phase, arterial phase (20–30 seconds after contrast injection), portal venous phase (60–70 seconds post-injection), and delayed phase (160–180 seconds post-injection).

Appendix S2

Image Segmentation and image preprocessing

One radiologist (H.T.S. with 10 years of experience in abdominal imaging) delineated the tumor on each image using ITK-SNAP software (version 4.0.2; http://www.itksnap.org) across three cohorts. In cases where segmentation was ambiguous, another senior radiologist (M.S.Z, with 31 years of expertise in pancreatic diseases) was consulted for an expert assessment. Two months later, MR images from 30 patients were randomly selected and re-segmented by (L.L.) in a blind fashion to assess inter-observer variability. An ICC value greater than 0.80 was deemed indicative of good reproducibility.

In the aspect of image pre-processing, considering the layer thickness and the difficulty it brought to registration, $T1_{Pre}$, $T1_{Arterial}$, $T1_{Venous}$, and $T1_{Delayed}$ sequences were utilized for clustering. To obtain a reliable habitat imaging in clinical settings, three-phase enhancement ratio mappings, including Enhanced Ratio Mapping_arterial (Enhanced_A), Enhanced Ratio Mapping_venous (Enhanced_V), Enhanced Ratio Mapping_delayed (Enhanced_D) were derived using the following equation: $Enhancement\ Ratio\ Mapping = \frac{T1_{Enhanced} - T1_{Pre}}{T1_{Pre}}$.

The arterial phase is considered the most prominent contrast-enhanced phase for tumor depiction. Consequently, the remaining phases, $T1_{Pre-contrast}$, $T1_{Venous}$, and $T1_{Delayed}$ were aligned to $T1_{Arterial}$ through SyN transform using the open-source Python registration package ANTsPy.

Appendix S3

Habitat Imaging and Radiomic Feature Extraction

Within the feature space, the K-means algorithm was used to conduct the habitat imaging as follows:

1. K data points are randomly selected as the initial cluster centroids.

$$\mu_j^{(0)} = (A_j, V_j, D_j)$$

   where A, V, D represent the enhancement mappings derived from arterial, venous, and delayed phases, respectively.

2. For each voxel $x_i = (A_i, V_i, D_i)$ within the tumor region, the distance to each centroid was calculated and was then assigned to the nearest centroid.

$$c(i) = \arg\min_j \sqrt{(A_i - A_j)^2 + (V_i - V_j)^2 + (D_i - D_j)^2}$$

3. According to the assignment results, new cluster centroids were recalculated.

$$\mu_j = \left(\frac{1}{|C_j|} \sum_{i \in C_j} A_i , \frac{1}{|C_j|} \sum_{i \in C_j} V_i , \frac{1}{|C_j|} \sum_{i \in C_j} D_i \right)$$

   where C_j is the set of all data in the j-th cluster, and $|C_j|$ is the number of data points in the j-th cluster.

4. Repeat step 2 and 3 until a stopping criterion is met. During iteration, the objective function is to minimize the sum of distances from each data point to its cluster centroid.

$$\mathcal{J} = \sum_{i=1}^{n} \sum_{j=1}^{K} r_{ij} \left[(A_i - A_j)^2 + (V_i - V_j)^2 + (D_i - D_j)^2 \right]$$

   where r_{ij} is an indicator variable that is 1 if data point x_i is in cluster j and 0 otherwise.

Since the K-means algorism was applied to the enhancement mappings which were derived from four original sequences, the sub-regions generated by the K-means clustering could match both three parametric mappings and

$T1_{Pre-contrast}$, $T1_{Arterial}$, $T1_{Venous}$, and $T1_{Delayed}$ phases. Subsequently, the enhance_habitat features and original_habitat features were extracted from these two groups of images, respectively. Additionally, as for the six original sequences, which encompass the previous four sequences along with DWI and T2 radiomics features were extracted to develop the radiomics model.

For each single ROI, a total of 2264 radiomics features were extracted utilizing uAI Research Portal (United Imaging Intelligence, China) including first-order (grayscale), second-order (shape), and texture features.

Appendix S4

Model Evaluation and evaluation

For clinical features, both univariate and multivariate logistic regression analyses were applied to select those features with a p-value of less than 0.05.

The logistic regression (LR) model typically allowed for relatively balanced performance across various datasets and are less prone to overfitting the training data. Therefore, the LR model was selected for conducting experiments. For the conventional radiomics model, the selected features from $T1_{Pre}$, $T1_{Arterial}$, $T1_{Venous}$, $T1_{Delayed}$, DWI, and $T2$ models were further selected by LASSO. The same process was also applied on the original_habitat and enhance_habitat models. Combine these three radiomics models to form an image model and then together with clinical model to construct the final combined model.

The receiver operating characteristic (ROC) curve and the corresponding the area under the curve (AUC) were calculated quantitatively. Additionally, five parameters, including accuracy (ACC), sensitivity, specificity, precision, and F1-score were calculated to quantitatively evaluate the consistency between the actual label and predictive label.

These five equations for model evaluations were defined as follows:

$$ACC = \frac{TP + TN}{TP + FP + TN + FN}$$

$$Sensitivity = Recall = \frac{TP}{TP + FP}$$

$$Specificity = \frac{TN}{FP + TN}$$

$$Precision = \frac{TP}{TP + FP}$$

$$F1\ score = \frac{2 \times Precision \times Recall}{Precision + Recall}$$

Table E1: Detailed parameters of MRI sequences in Center 1

Center	MRI	Parameters	TR	TE	FOV	Matrix	Section thickness(mm)	Gap
Zhongshan Hospital (Center 1)	1.5T MR (MAGNETOM Aera, Siemens)	T2-weighted imaging	4918	106	285×380	384×273	5.5	1.2
		DWI [a]	5100	55	285×380	192×154	5.5	1.2
		T1-weighted in-phase and opposed-phase imaging	6.88	2.39/4.77	356×380	320×240	3.5	0
		Contrast-enhanced T1-weighted imaging	3.47	1.36	308×380	320×240	3	0
	3.0T MR (Prisma, Siemens)	T2-weighted imaging	587	103	380×344	320×320	6	1.2
		DWI [a]	5000	65	380×310	130×130	5	1.2
		T1-weighted in-phase and opposed-phase imaging	3.97	1.29/2.52	380×285	320×288	3.0	0
		Contrast-enhanced T1-weighted imaging	3.10	1.23	380×309	320×288	3	0

Note. — TR = Repetition time; TE = Echo time; FOV = Field of view;
DWI = Diffusion-weighted Imaging;
[a] performed with b values of 0 and 500 sec/mm^2

Table E2: Detailed parameters of MRI sequences in multi-centers

Center	MRI	Parameters	TR	TE	FOV	Matrix	Section thickness(mm)	Gap
Affiliated Hospital of Jiangnan University (Center 2)	GE MEDICAL SYSTEMS SIGNA Architect 3.0T	T2-weighted imaging	10000	91.9	38×38	288×288	6.0	2.0
		DWI[b]	8000	68.4	38×38	128×130	6.0	2.0
		T1-weighted in-phase and opposed-phase imaging	4.8	2.2/1.1	38×30.4	288×224	2.5	0
		Contrast-enhanced T1-weighted imaging	4.8	1.7	38×30.4	288×224	2.6	0
	Philips Ingenia CX 3.0T	T2-weighted imaging	22552	89.1	40×40	288×58	6.0	1.0
		DWI[b]	14452	59.3	36.5×42.9	136×128	5.0	1.5
		T1-weighted in-phase and opposed-phase imaging	3.63	2.4/1.2	40×31.4	236×194	2.0	0
		Contrast-enhanced T1-weighted imaging	3.23	0.0	40×35.2	268×251	2.0	0

The First Hospital of Jiaxing (Center 3)	GE3.0T Discovery MR750	T2-weighted imaging	12000	80.5	40×40	352×352	6.5	1.0
		DWI [c]	6666.7	47.4	40×40	128×128	6.5	1.0
		T1-weighted in-phase and opposed-phase imaging	4	2.29/1.12	40×40	320×320	5	2.5
		Contrast-enhanced T1-weighted imaging	3.76	1.67	40×40	256×320	3	0
	Siemens Area1.5T	T2-weighted imaging	8941.1	85.0	35×35	256×256	6	1.2
		DWI [c]	5200	67	29.7×38	240×384	7	1.4
		T1-weighted in-phase and opposed-phase imaging	200	4.9/2.4	31.0×38.1	182×320	6.5	1.3
		Contrast-enhanced T1-weighted imaging	5.7	2.2	38×38	224×320	3	0
First Affiliated Hospital of Bengbu Medical University (Center 4)	Philips Ingenia CX 3.0T PHILIPS	T2-weighted imaging	1250	70	36.2×36.2	212×216	5	0.5
		DWI[c]	3000	59.3	37.5×37.5	132×161	6.5	3.25

	T1-weighted in-phase and opposed-phase imaging	165	2.3/1.15	34.8×34.8	152×194	7	0
	Contrast-enhanced T1-weighted imaging	3.03	1.42	34.5×34.5	232×264	4.6	0

Note. — TR = Repetition time; TE = Echo time; FOV = Field of view;
DWI = Diffusion-weighted Imaging;
[b] performed with b values of 0, 50, 800 sec/mm^2
[c] performed with b values of 0, 800 sec/mm^2

Table E3. The extracted radiomics features

Filter	Firstorder	Glcm	Gldm	Glrlm	Glszm	Ngtdm	Shape	Sum
Additivegaussiannoise	18	21	14	16	16	5		90
Binomialblurimage	18	21	14	16	16	5		90
Boxmean	18	21	14	16	16	5		90
Boxsigmaimage	18	21	14	16	16	5		90
Curvatureflow	18	21	14	16	16	5		90
Discretegaussian	18	21	14	16	16	5		90
Laplaciansharpening	18	21	14	16	16	5		90
Log	72	84	56	64	64	20		360
Mean	18	21	14	16	16	5		90
Normalize	18	21	14	16	16	5		90
Original	18	21	14	16	16	5	14	104
Recursivegaussian	18	21	14	16	16	5		90
Shotnoise	18	21	14	16	16	5		90
Specklenoise	18	21	14	16	16	5		90
Wavelet	144	168	112	128	128	40		720
Sum	450	525	350	400	400	125	14	2264

Table E4. The extracted habitat features

Filter	Firstorder	Glcm	Gldm	Glrlm	Glszm	Ngtdm	Shape	Sum
Additivegaussiannoise	18	21	14	16	16	5		90
Binomialblurimage	18	21	14	16	16	5		90
Boxmean	18	21	14	16	16	5		90
Boxsigmaimage	18	21	14	16	16	5		90
Curvatureflow	18	21	14	16	16	5		90
Discretegaussian	18	21	14	16	16	5		90
Laplaciansharpening	18	21	14	16	16	5		90
Mean	18	21	14	16	16	5		90
Normalize	18	21	14	16	16	5		90
Original	18	21	14	16	16	5	14	104
Recursivegaussian	18	21	14	16	16	5		90
Shotnoise	18	21	14	16	16	5		90
Specklenoise	18	21	14	16	16	5		90
Wavelet	144	168	112	128	128	40		720
Sum	378	441	294	336	336	105	14	1904

Table E5 Performance of Radiomics Models from Single Sequence

Models	AUC(95% CI)		Sensitivity		Specificity		Accuracy		Precision		F1 Score	
	Derivation	Interna validation	Derivation	Interna validation	Derivation	Interna validation	Derivation	Interna validation	Derivation	Interna validation	Derivation	Interna validation
Radiomics_DWI	0.681(0.631-0.731)	0.600(0.49-0.709)	0.753	0.638	0.451	0.581	0.581	0.606	0.509	0.536	0.607	0.583
Radiomics_T2	0.613(0.56-0.667)	0.674(0.573-0.776)	0.554	0.638	0.561	0.661	0.558	0.651	0.488	0.588	0.519	0.612
Radiomics_T1_Pre	0.638(0.585-0.69)	0.616(0.508-0.723)	0.398	0.447	0.756	0.742	0.602	0.615	0.552	0.568	0.462	0.5
Radiomics_T1_A	0.671(0.619-0.723)	0.605(0.498-0.712)	0.758	0.766	0.459	0.452	0.588	0.587	0.515	0.514	0.613	0.615
Radiomics_T1_V	0.668(0.617-0.719)	0.617(0.509-0.725)	0.489	0.489	0.748	0.726	0.637	0.624	0.595	0.575	0.537	0.529
Radiomics_T1_D	0.666(0.615-0.717)	0.650(0.545-0.754)	0.774	0.766	0.451	0.484	0.59	0.606	0.516	0.529	0.619	0.626

Table E6 Performance of Habitat Models from Different Sequence

Models	AUC(95% CI)		Sensitivity		Specificity		Accuracy		Precision		F1 Score	
	Derivation	Internal validation	Derivation	Internal validation	Derivation	Internal validation	Derivation	Internal validation	Derivation	Internal validation	Derivation	Internal validation
Habitat_T1_Pre_1	0.677(0.627-0.728)	0.589(0.479-0.698)	0.769	0.702	0.488	0.452	0.609	0.56	0.532	0.493	0.629	0.579
Habitat_T1_A_1	0.686(0.635-0.736)	0.553(0.444-0.662)	0.731	0.596	0.524	0.548	0.613	0.569	0.538	0.5	0.62	0.544
Habitat_T1_V_1	0.636(0.583-0.688)	0.424(0.315-0.532)	0.651	0.404	0.541	0.452	0.588	0.431	0.517	0.358	0.576	0.38
Habitat_T1_D_1	0.690(0.64-0.74)	0.447(0.337-0.558)	0.64	0.404	0.618	0.532	0.627	0.477	0.559	0.396	0.596	0.4
Habitat_T1_Pre_2	0.671(0.622-0.72)	0.504(0.397-0.61)	0.833	0.787	0.394	0.339	0.583	0.532	0.51	0.474	0.633	0.592
Habitat_T1_A_2	0.595(0.543-0.647)	0.496(0.39-0.601)	0.876	0.872	0.232	0.226	0.509	0.505	0.463	0.461	0.606	0.603
Habitat_T1_V_2	0.622(0.57-0.673)	0.574(0.469-0.68)	0.849	0.851	0.329	0.29	0.553	0.532	0.489	0.476	0.621	0.611
Habitat_T1_D_2	0.584(0.532-0.637)	0.492(0.386-0.598)	0.823	0.809	0.285	0.258	0.516	0.495	0.465	0.452	0.594	0.58
Habitat_T1_Pre_3	0.617(0.563-0.67)	0.532(0.421-0.642)	0.548	0.489	0.593	0.613	0.574	0.56	0.505	0.489	0.526	0.489
Habitat_T1_A_3	0.595(0.54-0.649)	0.436(0.327-0.545)	0.962	0.936	0.053	0.113	0.444	0.468	0.434	0.444	0.599	0.603
Habitat_T1_V_3	0.648(0.596-0.701)	0.643(0.539-0.748)	0.543	0.532	0.667	0.71	0.613	0.633	0.552	0.581	0.547	0.556
Habitat_T1_D_3	0.608(0.555-0.661)	0.540(0.43-0.651)	0.581	0.574	0.606	0.548	0.595	0.56	0.527	0.491	0.552	0.529

Habitat_Enhanced_A_1	0.539(0.485-0.593)	0.554(0.445-0.662)	0.866	0.872	0.183	0.274	0.477	0.532	0.445	0.477	0.588	0.617
Habitat_Enhanced_V_1	0.659(0.608-0.71)	0.554(0.445-0.663)	0.984	1	0.065	0.145	0.461	0.514	0.443	0.47	0.611	0.639
Habitat_Enhanced_D_1	0.663(0.611-0.715)	0.653(0.55-0.756)	0.651	0.617	0.606	0.629	0.625	0.624	0.555	0.558	0.599	0.586
Habitat_Enhanced_A_2	0.558(0.505-0.611)	0.532(0.425-0.639)	0.349	0.723	0.703	0.355	0.551	0.514	0.471	0.459	0.401	0.562
Habitat_Enhanced_V_2	0.548(0.495-0.601)	0.544(0.435-0.653)	0.339	0.255	0.687	0.839	0.537	0.587	0.45	0.545	0.387	0.348
Habitat_Enhanced_D_2	0.584(0.532-0.636)	0.572(0.466-0.678)	0.247	0.298	0.789	0.806	0.556	0.587	0.469	0.538	0.324	0.384
Habitat_Enhanced_A_3	0.554(0.498-0.609)	0.576(0.467-0.686)	0.559	0.574	0.508	0.581	0.53	0.578	0.462	0.509	0.506	0.54
Habitat_Enhanced_V_3	0.667(0.615-0.719)	0.507(0.397-0.617)	0.274	0.17	0.898	0.903	0.63	0.587	0.671	0.571	0.389	0.262
Habitat_Enhanced_D_3	0.592(0.537-0.647)	0.536(0.426-0.645)	0.774	0.787	0.309	0.371	0.509	0.55	0.459	0.487	0.576	0.602

Table E7 Performance comparison between different models

Models	Derivation cohort AUC *P* value*	Internal validation cohort AUC *P* value*	External validation cohort AUC *P* value*
Radiomics vs Original_habitat	0.883	0.883	0.234
Radiomics vs Enhanced_habitat	0.646	0.752	0.405
Radiomics vs Habitat	0.260	0.624	0.126
Radiomics vs Clinical	0.298	0.966	0.743
Radiomics vs RH	0.002	0.258	0.239
Radiomics vs RHC	<0.001	0.045	0.026
Original_habitat vs Enhanced_habitat	0.502	0.857	0.799
Original_habitat vs Habitat	0.069	0.270	0.746
Original_habitat vs Clinical	0.214	0.925	0.453
Original_habitat vs RH	0.003	0.165	0.618
Original_habitat vs RHC	<0.001	0.026	0.518
Enhanced_habitat vs Habitat	0.006	0.248	0.475
Enhanced_habitat vs Clinical	0.531	0.798	0.577
Enhanced_habitat vs RH	<0.001	0.139	0.871
Enhanced_habitat vs RHC	<0.001	0.022	0.373
Habitat vs Clinical	0.027	0.634	0.299
Habitat vs RH	0.024	0.410	0.201
Habitat vs RHC	<0.001	0.081	0.575
Clinical vs RH	<0.001	0.409	0.690
Clinical vs RHC	<0.001	0.041	0.134
RH vs RHC	<0.001	0.136	0.018

*The DeLong test was employed to compare the AUCs of the various models.

Table E8 The Detailed Features Description

Feature ID	Feature description
F1	Imaging-enlarged_Lymph_Nodes
F2	TB_class
F3	CA199_class
F4	Enhance_D_1_binomialblurimage_ngtdm_Contrast
F5	Enhance_D_1_normalize_firstorder_InterquartileRange
F6	Enhance_D_1_normalize_firstorder_Median
F7	Enhance_D_1_laplaciansharpening_glcm_InverseVariance
F8	T2_boxsigmaimage_glcm_InverseVariance
F9	T1_D_boxmean_glcm_Idn
F10	T1_D_wavelet_firstorder_wavelet-HHL-Median
F11	T1_D_wavelet_gldm_wavelet-LLL-SmallDependenceLowGrayLevelEmphasis
F12	T1_V_2_wavelet_firstorder_wavelet-LHH-InterquartileRange
F13	T1_V_2_wavelet_glcm_wavelet-LHH-DifferenceAverage
F14	T1_V_3_normalize_firstorder_Minimum
F15	T1_V_3_normalize_glszm_SmallAreaLowGrayLevelEmphasis
F16	T1_Pre_1_boxsigmaimage_firstorder_Kurtosis
F17	T1_Pre_1_wavelet_glszm_wavelet-HHH-GrayLevelVariance
F18	T1_Pre_1_normalize_glrlm_ShortRunLowGrayLevelEmphasis

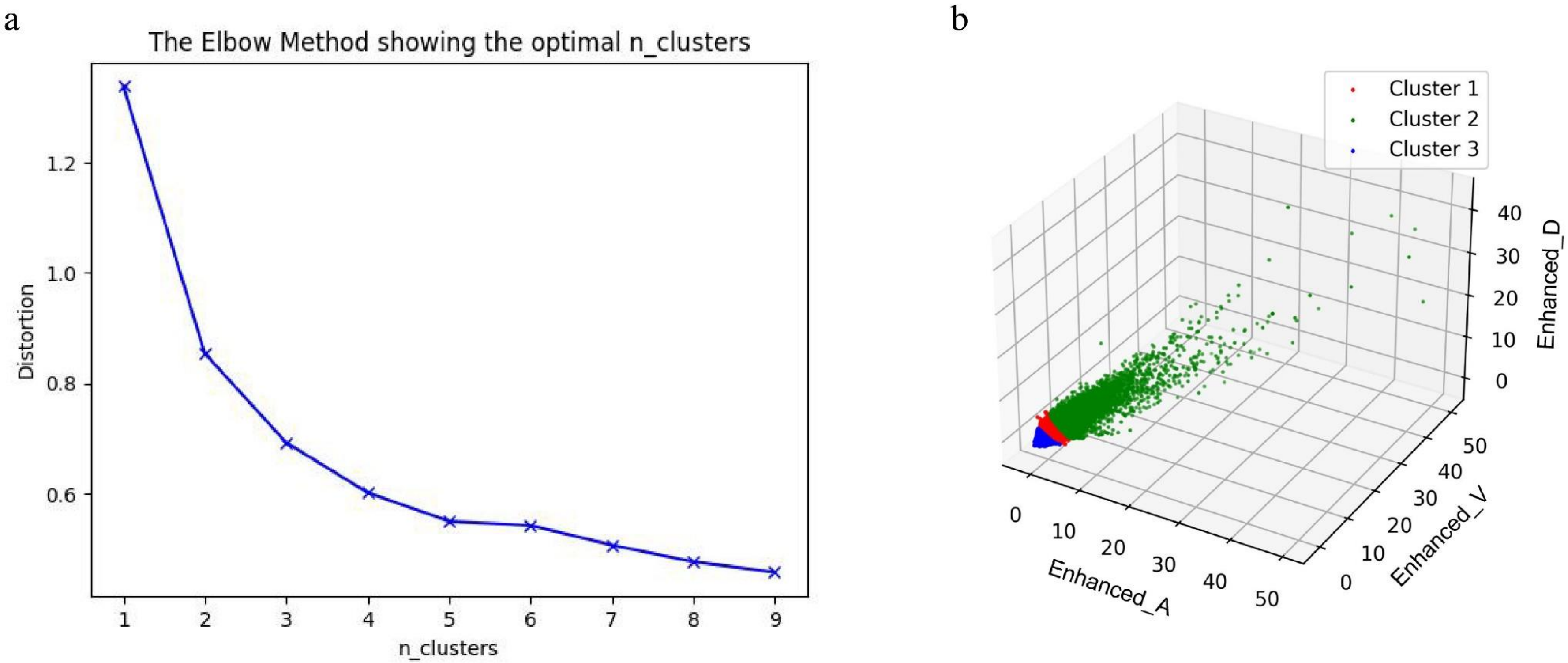

Figure E1 Habitat clustering results. (a) Calinski–Harabasz score line chart and (b) scatter plot of clustering analysis on enhancement ratio mapping.

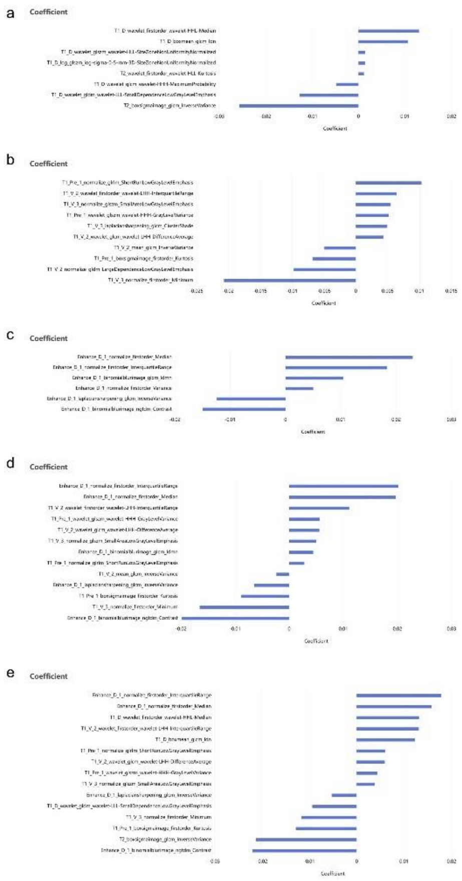

Figure E2. Selected features in five modality models. Features of (a) Radiomics model, (b) Origin_Habitat model, (c) Enhanced_Habitat model, (e) Habitat model, and (f) RH model were selected by K-best and LASSO regression.

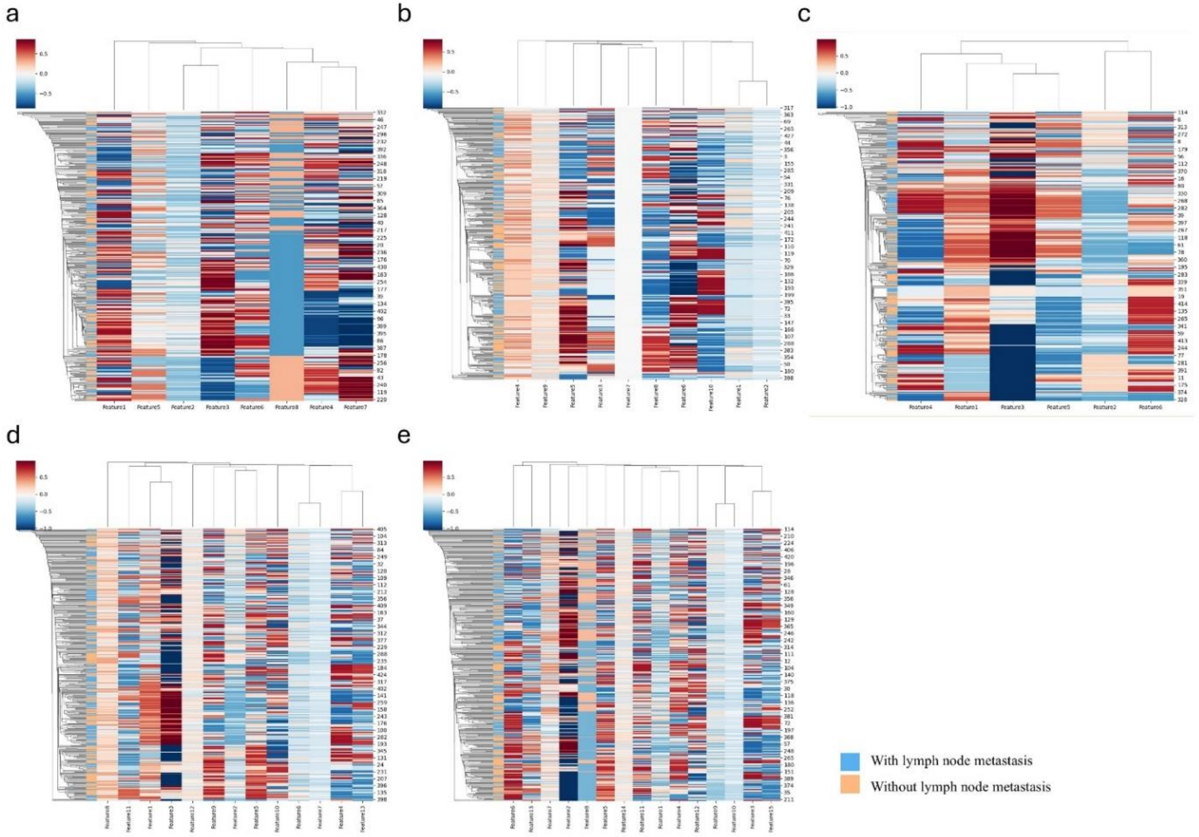

Figure E3. Clustering analysis for key radiomics and habitat features. Hierarchical clustering heatmap for key (a) radiomics features, (b) origin habitat features, (c) enhanced habitat features, (d) habitat features, and (a) radiomics-habitat (RH) features in the derivation cohort. The x-axis represents the IDs of radiomics and habitat features, and the y-axis represents patients. Patients belong to the same cluster (adjacent rows) share similar features in the Euclidean space. The status of lymph node metastasis (LNM) is displayed on the blue-orange bar located on the left side next to the y-axis.

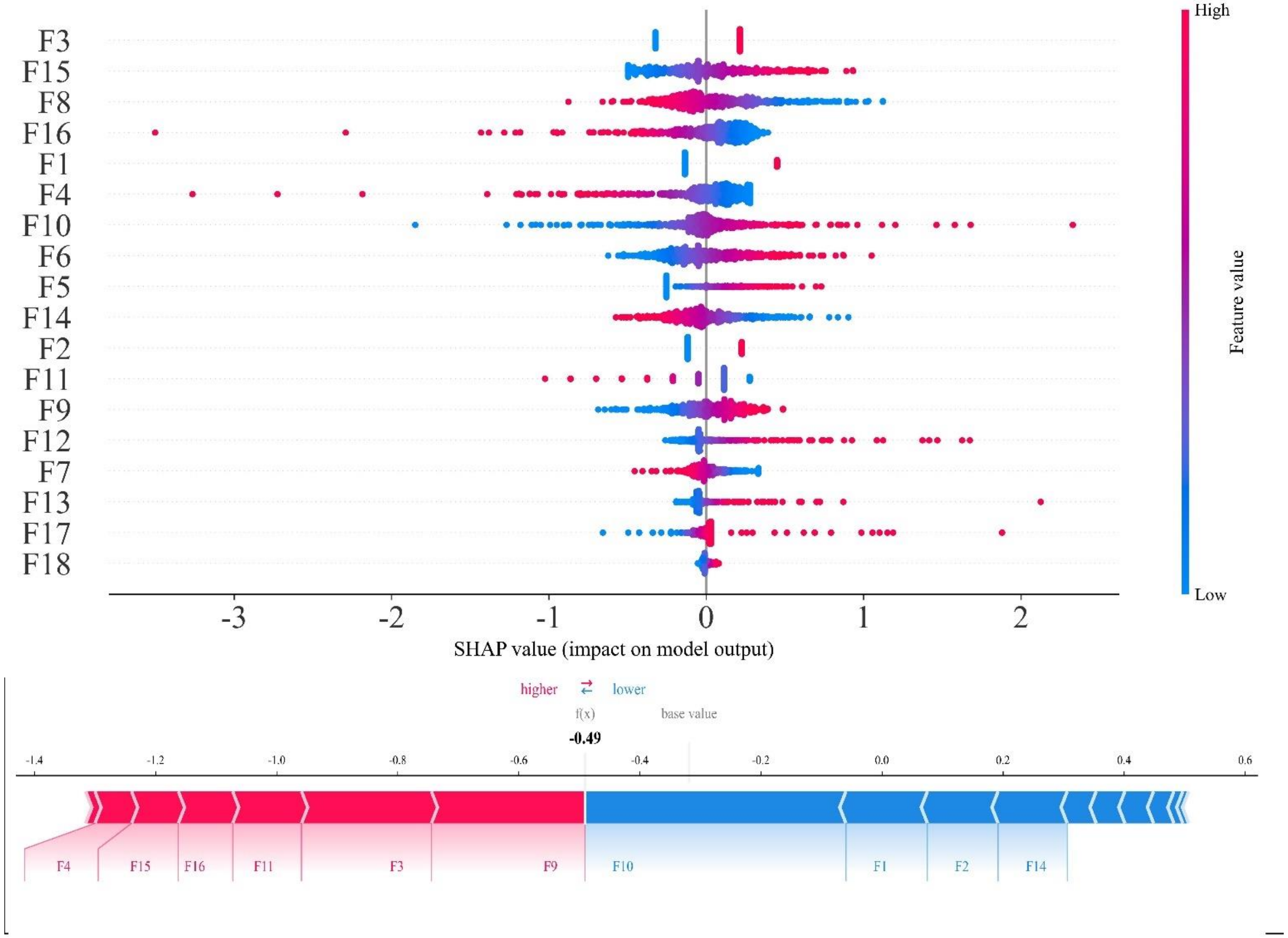

Figure E4. The SHapley Additive exPlanations (SHAP) for the RHC model.
